# Cardiotrophin-1 Induces Tumor Necrosis Factor *α* Synthesis in Human Peripheral Blood Mononuclear Cells

**DOI:** 10.1155/2009/489802

**Published:** 2010-03-10

**Authors:** Michael Fritzenwanger, Katharina Meusel, Christian Jung, Marcus Franz, Zhenhua Wang, Martin Foerster, Hans-R. Figulla

**Affiliations:** ^1^Division of Cardiology, Department of Internal Medicine I, Friedrich-Schiller-University of Jena, Erlanger Allee 101, 07740 Jena, Germany; ^2^Department of Cardiology, Second Affiliated Hospital of Fujian Medical University, Zhongshan North Road 34, Quanzhou, 362000 Fujian, Germany

## Abstract

Chronic heart failure (CHF) is associated with elevated concentrations of tumor necrosis factor (TNF) *α* and cardiotrophin-1 (CT-1) and altered peripheral blood mononuclear cell (PBMC) function. Therefore, we tested whether CT-1 induces TNF*α* in PBMC of healthy volunteers. CT-1 induced in PBMC TNF*α* protein in the supernatant and TNF*α* mRNA in a concentration- and time-dependent manner determined by ELISA and real-time PCR, respectively. Maximal TNF*α* protein was achieved with 100 ng/mL CT-1 after 3–6 hours and maximal TNF*α* mRNA induction after 1 hour. ELISA data were confirmed using immunofluorescent flow cytometry. Inhibitor studies with actinomycin D and brefeldin A showed that both protein synthesis and intracellular transport are essential for CT-1 induced TNF*α* expression. CT-1 caused a dose dependent nuclear factor (NF) *κ*B translocation. Parthenolide inhibited both NF*κ*B translocation and TNF*α* protein expression indicating that NF*κ*B seems to be necessary. We revealed a new mechanism for elevated serum TNF*α* concentrations and PBMC activation in CHF besides the hypothesis of PBMC activation by bacterial translocation from the gut.

## 1. Introduction

CHF is not only the failure of the heart to generate sufficient cardiac output, but is a multisystemic disorder with immune activation leading to increased concentrations of several cytokines [1].

In CHF several studies showed increased concentrations of proinflammatory cytokines such as TNF*α*, interleukin (IL)-1, IL-6, IL-18, and cardiotrophin-1 (CT-1) [2–5]. One of the most examined proinflammatory molecules in CHF is TNF*α*. TNF*α* is a trimeric 17-kDa polypeptide mainly produced by monocytes and macrophages. The effects of TNF*α* on cardiac function are concentration and time-dependent. Short-term TNF*α* expression is thought to be an adaptive mechanism; whereas prolonged expression causes left ventricular dysfunction and cardiomyopathy leading to CHF propagation. However, TNF*α* influences not only the heart itself but causes endothelial dysfunction and peripheral muscle wasting [6].

Cardiotrophin-1 (CT-1) is a member of the IL-6 cytokine family that consists of IL-6, IL-11, ciliary neurotrophic factor (CNTF), cardiotrophin-1 (CT-1), cardiotrophin-like cytokine (CLC), leukemia inhibitory factor (LIF), neuropoietin (NPN), and oncostatin M (OSM) and has recently been supplemented by the addition of two newly characterized cytokines: IL-27 and IL-31 [7]. All these cytokines bind to a specific receptor chain (IL-6R, IL-11R, or LIFR for CT-1, LIF, OSM). Following cytokine binding the cytokine/receptor complex associates with glycoprotein 130 (gp130) causing tyrosine phosphorylation of gp130 and the signal is transduced via the Janus kinase (JAK)/signal transducer and activation of transcription 3 (STAT3) pathway [8–10]. CT-1 is expressed in a time-dependent manner during embryogenesis of organs, is expressed in the heart during life, induces cardiac myocyte hypertrophy, and is able to prevent myocyte apoptosis via a mitogen dependent kinase pathway [8, 11]. 

Increased CT-1 concentrations were detected in patients with acute myocardial infarction and chronic heart failure (CHF). Furthermore, CT-1 plasma concentrations correlate with the severity of left ventricular dysfunction [11–14]. However, CT-1 has not only effects on myocytes but also on vasculature by decreasing systemic vascular resistance in an animal model [15], by induction of acute phase proteins in rat hepatocytes [16], and by attenuation of endotoxin-induced acute lung injury [17]. 

There are several studies showing that in CHF PBMCs produce TNF*α* [18, 19]. But so far the mechanisms responsible for TNF*α* production in these cells under these circumstances are not determined. 

In this study we investigated whether CT-1 induces TNF*α* expression in human PBMC of healthy volunteers. Furthermore, we designed inhibitor experiments to characterise the underlying pathway.

## 2. Materials and Methods

### 2.1. Reagents

Recombinant human CT-1 was purchased from R&D Systems (Wiesbaden, Germany) and dissolved according to the manufacturer's instruction. Actinomycin D, brefeldin A, and parthenolide were purchased from Sigma Chemicals (Deisenhofen, Germany). The blocking antibody against CT-1 was purchased from R&D Systems (Wiesbaden, Germany).

### 2.2. Cell Culture

Human peripheral blood mononuclear cells were obtained from healthy volunteers by Ficoll-paque (Amersham Bioscience, Uppsala, Sweden) centrifugation. The cells were washed three times with PBS, resuspended in RPMI 1640 supplemented with 10% fetal calf serum, 1% penicillin, streptomycin (all from Biochrom AG, Berlin, Germany), and cultured in plastic dishes at 37°C  in a humified 5% CO_2_ atmosphere. Cells were cultivated for 24 hour with RPMI 1640 supplemented with 10% fetal calf serum, 1% penicillin, streptomycin. Afterwards, cells were subconfluent and medium was replaced by fresh medium. After 24 hours, over 90% of PBMC were alive tested by trypan blue exclusion. Stimulation and pharmacological studies were done afterwards. 

Primary cultures from human vein endothelial cells were purchased from PromoCell (Heidelberg, Germany). Cell culture was done according to the manufacturer's manual in endothelial growth medium with 2% fetal calf serum (EGM, PromoCell, Heidelberg, Germany). Cells were grown to confluence in collagen I coated tissue culture plastic (Becton Dickinson, Franklin Lakes, USA). Cells were used in the second to fifth cell passages.

All stimulants, inhibitors and media were without significant endotoxin levels according to the manufacturers' instructions.

Pharmacological agents, dissolved in fresh medium, were added to the cells for defined time intervals and concentrations. As a control, fresh medium was added to the cells.

Approval for this study was given by the Ethics Committee of the Friedrich Schiller University of Jena, and subjects gave their written informed consent according to the university guidelines.

### 2.3. Real-Time PCR

Total RNA from cultivated PBMC was extracted according to the RNeasy protocol (Qiagen, Hilden, Germany). One *μ*g of total RNA was reversely transcribed into cDNA in a volume of 20 *μ*l with avian myeloma leukaemia virus (AMV) reverse transcriptase and oligo dT primers (Promega, Madison, USA) according to the manufacturers manual.

Real-time PCR measurement of TNF*α* cDNA was performed with the Light Cycler Instrument using the Fast Start DNA Master SYBR Green I kit (Roche Diagnostics, Mannheim, Germany). For verification of the correct amplification product, PCR products were analyzed on a 2% agarose gel stained with ethidium bromide. The specific primer for human TNF*α* was purchased from R&D Systems. The amplification program for TNF*α* consisted of 1 cycle of 94°C with a 4-minute hold followed by 40 cycles of 95°C with a 45-second hold, 59°C annealing temperature with a 45-second hold, and 72°C with a 45-second hold. The specific primer pair for GAPDH was: sense primer 5′ GGG AAG GTG AAG GTC GG 3′, antisense primer 5′ TGG ACT CCA CGA CGT ACT CAG 3′. The amplification program for GAPDH consisted of 1 cycle of 95°C with a 30-second hold followed by 30 cycles of 95°C with a 5-second hold, 59°C annealing temperature with a 10-second hold, and 72°C with a 20-second hold. Each reaction (20 *μ*l) contained 2 *μ*l cDNA, 2.5 mM MgCl_2_, 1 pmol of each primer, and 2 *μ*L of Fast Starter Mix (containing buffer, dNTPs, Sybr Green dye and Taq polymerase). Amplification was followed by melting curve analysis to verify the correctness of the amplicon. A negative control without cDNA was run with every PCR to assess the specificity of the reaction. Analysis of data was performed using Light Cycler software version 3.5. PCR efficiency was determined by analysing a dilution series of cDNA (external standard curve). The identity of the PCR product was confirmed by comparing its melting temperature (Tm) with the Tm of amplicons from standards or positive controls. GAPDH was analyzed in parallel to each PCR and the resulting GAPDH values were used as standards for presentation of TNF*α* transcripts.

### 2.4. TNF*α* ELISA

Cultured PBMCs were treated with various concentrations of CT-1 for various time periodes. TNF*α* concentrations in the culture supernatants were determined by ELISA (QuantiGlo, R&D Systems, Wiesbaden, Germany) according to the manufacturer's instructions.

### 2.5. EMSA

Nuclear extracts were achieved by the EpiQuik Nuclear Extraction KIT I (Epigentek, NY, USA) according to the manufacturer's manual. Afterwards, protein concentrations of nuclear extracts were determined according to the Bradford methode. For determination of NF*κ*B 2 *μ*g of nuclear proteins were used and further analyzed by gel electrophoretic mobility shift assay (EMSA) according to the suppliers manual. EMSA kits and probes were purchased from Panomics, Redwood City, USA. 

### 2.6. Immunofluorescent Flow Cytometric Analysis of Cytokine Production

For intracellular staining peripheral blood was collected in lithium-heparin tubes. 100 *μ*l of blood was added to RPMI-1640 medium including brefeldin A (final concentration: 1 *μ*g/ml) (Sigma, Taufkirchen, Germany), and incubated for 6 hours time at 37°C. Next, erythrocytes were lysed by NH_4_Cl. After washing with PBS/2% FCS cells were stained with monoclonal antibodies against the surface antigens CD3 (Coulter-Immunotech, Krefeld, Germany), CD4 (Caltag, Hamburg, Germany) CD8 or CD14 (BD-Pharmingen, Heidelberg, Germany) (15 minute, RT), followed, after a washing step, by fixation with 100 *μ*l 2% paraformaldehyde for 10 min at RT. After a wash the cells were incubated in 100 *μ*l permeabilisation solution (0,1% saponin and 0,1% NaN_3_ in PBS) together with 1 *μ*l directly conjugated anti-TNF*α* antibody (BD-Pharmingen, Heidelberg, Germany) for 15 minute at RT. Followed by a wash with permeabilisation solution the cells were resuspended in PBS/2% FCS and fluorescence intensity was analyzed by flow cytometry (FACSCalibur, Becton-Dickinson, Heidelberg, Germany). For analysis regions were defined by forward scatter and side scatter as well as CD3^+^/CD4^+^- or CD3^+^/CD8^+^-lymphocyte populations and CD14^+^ monocyte population. Data were analyzed with CellQuest Software.

### 2.7. Statistical Analysis

Because the amount of the cytokines produced was different in each experiment, the effects on TNF*α* production were normalized to unstimulated cells, which were set as one. Data were analysed by nonparametric methods to avoid assumptions about the distribution of the measured variables. Comparisons between groups were made with the Wilcoxon test. All values are reported as means  ±  SEM. Statistical significance was considered to be indicated by a value of *P* < .05.

## 3. Results

### 3.1. CT-1 Induces TNF*α* Protein and mRNA Levels in PBMC

**Figure 1:**
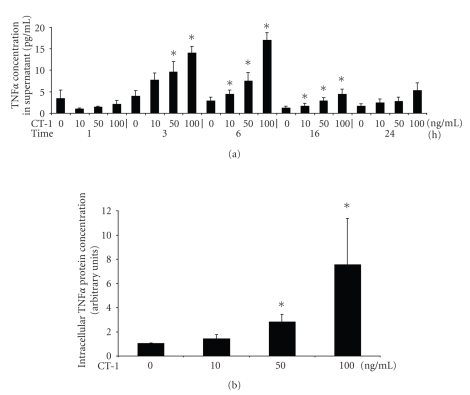
(a) Concentration- and time-dependent expression of TNF*α* protein in the supernatant after incubation with CT-1. Human PBMCs were incubated with various concentrations of CT-1 and for different periodes. After the indicated time TNF*α* protein concentration was determined by a commercial available ELISA. *n* = 5, data are expressed as mean  ±  SEM. *P < .05 compared to unstimulated cells. (b) Analysis of intracellular TNF*α* production using immunofluorescent flow cytometry. Human blood was incubated with different concentrations of CT-1 for 6 hours in the presence of brefeldin A. Afterwards erythrocytes were lysed and cells were stained with a monoclonal antibody against CD14 FITC-conjugated and against TNF*α* PE-conjugated. Monocytes were gated and results are expressed normalized to unstimlulated monocytes. *n* = 11, data are expressed as mean  ±  SEM. *P < .05 compared to unstimulated cells.

In the first sets of experiments we analysed whether CT-1 is able to induce TNF*α* in PBMC. As shown in Figure 1(a), CT-1 induced in a concentration-dependent manner TNF*α* in the supernatant determined by a commercial available ELISA. Maximal TNF*α* concentration was found after 3 to 6 hours and declined afterwards reaching nearly control values after 24 hours, indicating that CT-1 causes only a transient TNF*α* release in PBMC. In the next experiments we determined intracellular TNF*α* protein in monocytes, CD4^+^ and CD8^+^ lymphocytes after stimulation with various concentrations of CT-1 in the presence of brefeldin A using immunofluorescent flow cytometry. Intracellular TNF*α* determination in CD4^+^ and CD8^+^ lymphocytes did not show an effect of CT-1 on TNF*α* expression (data not shown). In monocytes we found a concentration-dependent increase of intracellular TNF*α* after CT-1 application (Figure 1(b)). These results showed that CT-1 induced TNF*α* in PBCM independent of culture conditions and independent of determination methodes.

**Figure 2:**
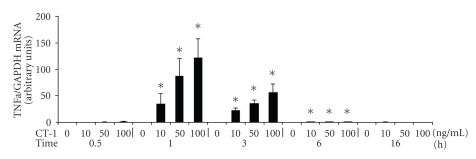
Concentration- and time-dependent induction of TNF*α* mRNA after incubation with CT-1. Human PBMCs were incubated with various CT-1 concentrations and for various periodes. After the indicated time mRNA was determined by real-time PCR. All TNF*α* mRNA expression data were normalized to GAPDH. n = 8, data are expressed as mean  ±  SEM. *P < .05 compared to unstimulated cells.

On TNF*α* mRNA level we found maximal mRNA after 1 hour. Afterwards TNF*α* mRNA decreased (Figure 2). Blocking CT-1 by an antibody against CT-1 inhibited CT-1 induced TNF*α* mRNA (data not shown) indicating that TNF*α* induction is specifically caused by CT-1.

### 3.2. The Effect of CT-1 on TNF*α* Expression in PBMC Is Dependent on mRNA Synthesis and Intracellular Protein Transport

**Figure 3:**
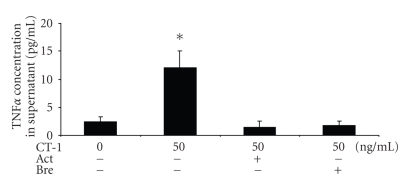
After 3 hours TNF*α* protein was determined by ELISA in the supernatant. CT-1 0 ng/mL was set as 1. Act: actinomycin D (5 *μ*g/mL), inhibits mRNA transcription, Bre: brefeldin (10 *μ*g/ml), inhibits intracellular protein transport. *n* = 6, data are expressed as mean  ±  SEM. *P < .05 compared to unstimulated cells.

With the next experiments we addressed the question whether TNF*α* protein expression is dependent on mRNA synthesis and intracellular protein transport. In Figure 3 it is shown that both inhibition of mRNA synthesis by actinomycin D and intracellular protein transport by brefeldin A were able to abolish CT-1 induced TNF*α* protein induction in the supernatant. These results showed that CT-1 was responsible for new protein synthesis of TNF*α* protein. Furthermore TNF*α* protein was secreted into supernatant actively.

### 3.3. CT-1 Induces TNF*α* via NF*κ*B

**Figure 4:**
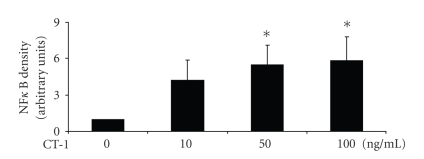
CT-1 causes a concentration dependent increase of NF*κ*B activity measured by EMSA. Bands corresponding to NF*κ*B activity were quantified by densitometry and expressed in arbitrary units and normalized to unstimulated PBMC. *n* = 7, data are expressed as mean  ±  SEM. *P < .05 compared to unstimulated cells.

As shown in Figure 4 CT-1 caused a concentration-dependent NF*κ*B translocation to the nucleus determined by EMSA reaching maximal translocation with 100 ng/ml CT-1.

**Figure 5:**
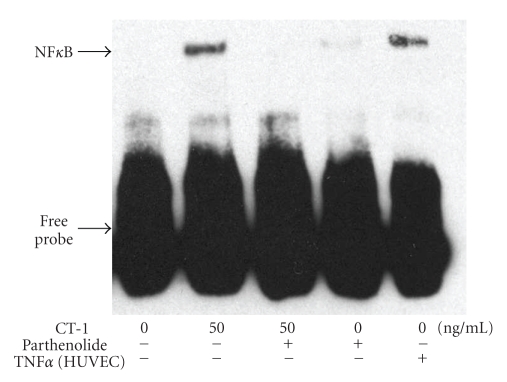
Detection of NF*κ*B activity in human PBMC. Representative EMSA of CT-1 induced NF*κ*B activity in PBMC, which could be inhibited by parthenolide an inhibitor of NF*κ*B activation. Human umbilical vein endothelial cells (HUVECs) stimulated with TNF*α* were used as control.

In the next sets of experiments we used EMSA to verify that NF*κ*B activation was responsible for CT-1 induced TNF*α* expression in PBMC. Human umbilical vein endothelial cells (HUVECs) stimulated with TNF*α* were used as a control. Unstimulated cells did not show significant NF*κ*B protein in the nucleus; whereas CT-1 caused translocation of NF*κ*B into the nucleus. Parthenolide, an inhibitor of NF*κ*B activation, was able to inhibit NF*κ*B translocation to the nucleus (Figure 5).

**Figure 6:**
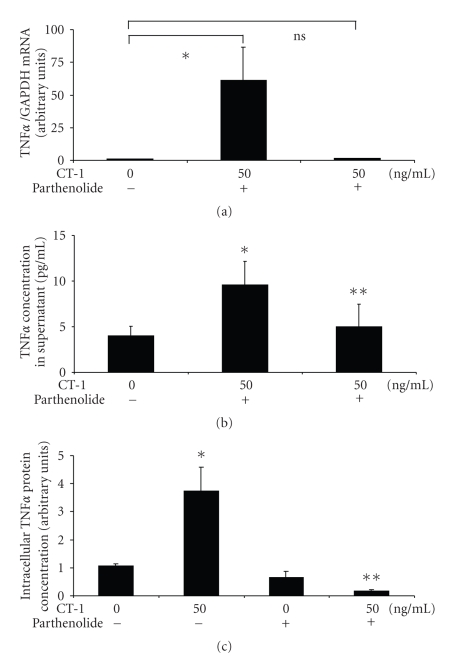
Effect of parthenolide on CT-1 induced TNF*α* expression. (a) Human PBMCs were incubated with CT-1 (50 ng/ml) for 1 hour in the presence of parthenolide. Afterwards cells were lysed and TNF*α* mRNA expression was determined by real-time-PCR. All TNF*α* mRNA expression data were normalized to GAPDH. *n* = 6, data are expressed as mean  ±  SEM. *P < .05 compared to unstimulated cells. (b) Human PBMCs were incubated with CT-1 (50 ng/ml) for 3 hours in the presence of parthenolide. Afterwards TNF*α* protein concentration in the supernatant was determined by ELISA. *n* = 5, data are expressed as mean  ±  SEM and normalized to unstimulated cells. *P < .05 compared to unstimulated cells. **not significant compared to unstimulated cells. (c) Analysis of intracellular TNF*α* production using immunofluorescent flow cytometry. Human blood was incubated with 50 ng/ml CT-1 for 6 hours in the presence of brefeldin A and parthenolide. Afterwards erythrocytes were lysed and cells were stained with a monoclonal antibody against CD14 FITC-conjugated and against TNF*α* PE-conjugated. Monocytes were gated and results are expressed normalised to unstimlulated monocytes. n=6, data are expressed as mean  ±  SEM. *P < .05 compared to unstimulated cells, **P < .05 compared to cells stimulated with 50 ng/ml CT-1.

NF*κ*B translocation is essential for TNF*α* expression as shown in Figure 6(a) and 6(b). Because parthenolide was able to inhibit TNF*α* expression both on protein and mRNA level we conclude that CT-1 not only was responsible for NF*κ*B translocation to the nucleus but this translocation was responsible for TNF*α* expression. Using flow cytometry we found in monocytes an increase of intracellular TNF*α* after CT-1 application which could be inhibited by parthenolide (Figure 6(c)). Parthenolide alone did not show a significant effect on TNF*α* expression in unstimulated cells. These results show that CT-1 induced TNF*α* in PBCM independent of culture conditions and independent of determination methodes and NF*κ*B seems to be essential for the effect of CT-1 on TNF*α* induction in PBMC.

## 4. Discussion

The first result of our study is that CT-1 is able to induce TNF*α* mRNA and protein in PBMC of healthy volunteers. 

TNF*α* is increased in serum of patients with CHF and correlates with the severity of heart failure, cachexia [20], and clinical outcome [21]. TNF*α* may be involved in progression of CHF because high levels of TNF*α* can induce left ventricular dysfunction, ventricular remodelling, cardiomyopathy, and pulmonary edema [22, 23]. 

Cultured human PBMC can synthesize and secrete TNF*α*. In heart failure, both the heart itself and activated monocytes are able to secrete TNF*α* [18, 24]. Furthermore, the capacity of PBMC of CHF patients to secrete TNF*α* is increased compared to control. Our data are in good agreement with these former studies and in opposite to Shimokawa et al. [25] who found decreased cytokine generation capacity of monocytes in severe heart failure after stimulation with lipopolysaccharide.

Besides the hypothesis that in CHF the failing heart itself is the main source of TNF*α* it is speculated by other groups that activated monocytes are responsible for increased TNF*α* serum concentrations. Monocytes may be activated by LPS from the gut because the barrier function of the gut by cardial edema is disturbed and bacteria can easily translocate from the gut lumen to the blood stream [26]. 

As a third possibility our data suggest at least in theory a new mechanism for TNF*α* production of PBMC in heart failure. CT-1 produced by the failing ventricle [27] is able to induce TNF*α* in PBMC without LPS. The here presented mechanism might also explain why TNF*α* may be still elevated in CHF even after edema were treated successfully with diuretics and the integrity of gut mucosa was restored. Furthermore, our data support the study of Petretta et al. [28] who found that TNF*α* is not produced by the failing heart or the gut in patients with mild to severe heart failure. 

The second result of our study is the fact that CT-1 activates the NF*κ*B system in a concentration-dependent manner in PBMC of healthy volunteers. Our in vitro data are in line with studies that found an activation of the NF*κ*B system in peripheral blood cells in CHF. Jankowska et al. reported an activation of the NF*κ*B system in peripheral blood leukocytes in CHF patients measured by immuncytochemistry [29]. Siednienko et al. found an augmented activation of NF*κ*B activation in blood mononuclear cells using electromobility shift assay in patients with CHF compared to healthy controls [30]. The exact pathway responsible for NF*κ*B activation in CHF is still unknown and remains to be determined. 

Our study has several limitations. We only used inhibitor experiments to characterise the pathway responsible. Furthermore we used a relative high parthenolide concentration. But within 3 hours, there is no cytotoxic effect as shown by O'Neill et al. in [31]. We also used high CT-1 concentrations compared to concentrations reported in patients with CHF by Ng et al. [12]. On the other hand a paper published in 2008 [32] reported serum CT-1 concentration in healthy controls and patients with metabolic syndrome of about 100 ng/ml. So far serum concentration of CT-1 in healthy controls and patients is a matter of discussion. But independent of reported CT-1 serum concentration the concentration of CT-1 should be much higher in the myocardium which is the source of CT-1 in CHF [33]. Exact intramyocardial CT-1 concentrations are not determined so far, only mRNA and immunohistochemical studies showed increased CT-1 in hearts of patients with CHF [34].

In our experiments both TNF*α* mRNA expression and TNF*α* protein production of PBMC showed a large standard variation. First one explanation for the large standard deviation may be a different genetic susceptibility of PBMC from different persons to stimuli [35]. Second, we used the low basal mRNA concentration as the basis of normalization explaining the large standard variation. Third, the fact that the increase of TNF*α* mRNA expression after CT-1 application is much higher compared to the increase of protein in the supernatant may be explained methodically. 

We used PBMC of healthy volunteers to examine the effect of CT-1 in CHF. Because in CHF many proinflammatory cytokines are elevated and PBMC are activated, it is not easy to study the effect of a single cytokine in PBMC of patients with CHF. For this reason we used PBMC from healthy volunteers in culture and stimulated them with recombinant CT-1.

In conclusion, our study offers a new mechanism of increased serum TNF*α* concentrations in heart failure. Interestingly, in our study LPS is not needed for elevated TNF*α* expression in PBMC. Elevated TNF*α* concentrations may be important in the pathogenesis and perpetuation of heart failure by modulating systemic metabolism, causing apoptosis and having a negative inotropic effect [36]. In the light of our results modulating CT-1 may be an interesting pharmacological target in the treatment of CHF.
